# Increased intraoperative vein ligation in microsurgical varicocelectomy is associated with pain improvement

**DOI:** 10.1097/MD.0000000000035170

**Published:** 2023-09-22

**Authors:** Wei-Chun Huang, Chi-Ping Huang, Chun-Ming Lai, Fang-Yu Ku, Hsu-Ning Hsu, Chao-Tung Yang, Yun-Yi Wang, Chun-Yo Laih

**Affiliations:** a Department of Urology, China Medical University Hospital, Taichung, Taiwan; b School of Medicine, China Medical University, Taichung, Taiwan; c Department of Computer Science, Tunghai University, Taichung, Taiwan; d GU plus urology clinic, Taipei, Taiwan; e Department of Gynecology and Obstetrics, China Medical University Hospital, Taichung, Taiwan.

**Keywords:** microsurgical varicocelectomy, varicocele pain, varicocele vein ligation, original study

## Abstract

Varicocele is a major cause of male infertility. However, few studies have discussed the potential associations between the pain caused by varicocele and preoperative and intraoperative factors. The aim of this study was to evaluate factors potentially associated with changes in pain score after microsurgical varicocelectomy. This retrospective study was conducted between August 2020 and August 2022 at China Medical University Hospital in Taichung, Taiwan. Patient characteristics including age, body mass index, semen analysis, testicular volume, and the number of veins ligated were collected. Preoperative and intraoperative factors were analyzed to determine if they were correlated with changes in numeric rating scale (NRS) after microsurgical varicocelectomy. A total of 44 patients with clinical varicocele underwent subinguinal microsurgical varicocelectomy and were analyzed. The overall pain resolution rate was 91%, and the average satisfaction score after surgery was 9.2 according to their subjective feelings. Multivariate analysis revealed that severe varicocele grade (odds ratio [OR] 16.5, 95% confidence interval [CI] 3.01–90.47; *P* = .018) and the number of veins ligated (OR 6, 95% CI 1.6–22.48; *P* = .013), were significantly associated with changes in NRS after surgery. In addition, the area under the receiver operating characteristic curve for changes in NRS and the total number of veins ligated was 0.869. Microsurgical varicocelectomy had a high success rate for scrotal pain and satisfaction. Severe varicocele grade and the number of veins ligated in microsurgical varicocelectomy were associated with postoperative pain improvement.

## 1. Introduction

Varicocele is an enlarged and tortuous pampiniform venous plexus in the spermatic cord which commonly causes scrotal pain.^[1]^ Most research on varicocele has focused on the link between varicocele and infertility, however, a significant number of patients come to our clinic seeking help because of varicocele-related discomfort. The pain caused by varicocele is characterized as a dull, throbbing, or aching sensation in the groin, scrotum, or testicle. Scrotal heaviness that intensifies with exercise or extended periods of standing can also be described as a varicocele.^[2]^

The etiology of scrotal pain associated with varicocele is not fully understood. Previous studies have mentioned possible mechanisms, such as compression of adjacent neural fibers by a dilated venous complex, increased scrotal temperature, oxidative stress to the testicular parenchyma, and tissue ischemia resulting from venous stasis.^[1,3]^

Conservative treatments, including anti-inflammatory drugs, scrotal support, and decreased physical activity are usually first-line therapies for varicocele-related discomfort.^[4,5]^ Varicocelectomy is indicated when conservative treatments fail.^[6]^ However, according to prior literature, up to 20% of individuals who undergo varicocelectomy may experience only partial or no improvement after surgery.^[6]^ Many studies conducted over the past 20 years have documented the advantages of varicocelectomy for uncomfortable varicoceles.^[6–8]^ Several studies have investigated whether clinical characteristics can predict surgical outcomes, with controversial findings. Some studies have reported that varicocele grade, pain quality and severity can predict favorable results,^[6–9]^ whereas others have found no correlation.^[10,11]^ Therefore, the aim of this study was to assess the efficacy of microsurgical varicocelectomy for treating scrotal pain and reevaluate the factors potentially related to changes in the patient pain score after microsurgical varicocelectomy.

## 2. Methods

**Figure 1.**
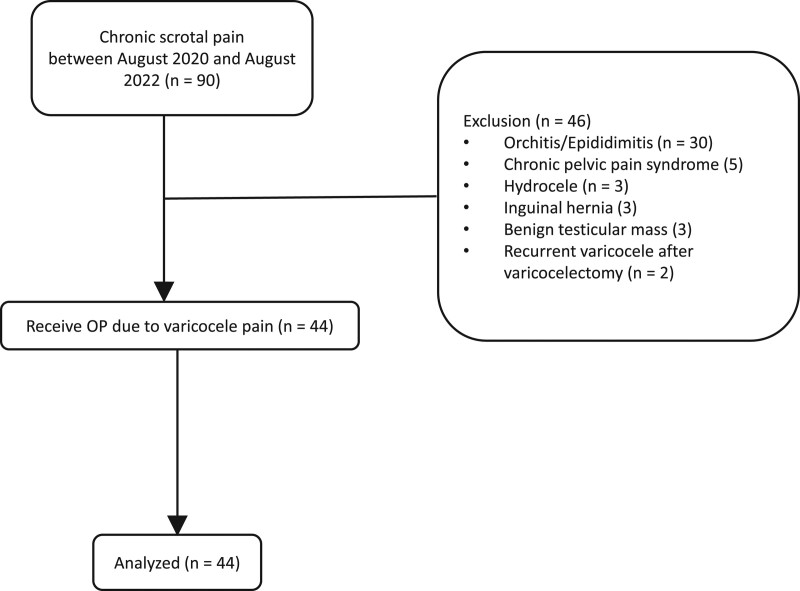
Patient enrollment.

This retrospective study was conducted between August 2020 and August 2022 at China Medical University Hospital in Taichung, Taiwan, during which 90 patients came to our clinic due to chronic scrotal pain. We excluded patients with other etiologies of scrotal pain, including orchitis, epididymitis, chronic pelvic pain syndrome, hydrocele, inguinal hernia, benign testicular mass, and recurrent varicocele after varicocelectomy. The remaining 44 patients received microsurgical subinguinal varicocelectomy and were enrolled in this study (Fig. 1). The patients’ demographic characteristics, including age, body mass index, semen analysis, testicular volume, varicocele location, varicocele grade and impaired semen analysis were collected (Table 1). The diagnosis and grading of the varicocele were conducted by a single experienced male infertility specialist according to the Dubin–Amelar criteria.^[12]^ Multivariate analysis was performed on the preoperative and intraoperative factors potentially associated with changes in the numeric rating scale (NRS) score and collected as our primary outcome.

**Table 1:** Demographic data and clinical characteristics of the patients.

Characteristic	Value
Age	33.3 ± 8.5
Height (cm)	173.1 ± 5.7
BW (kg)	72.5 ± 11.5
BMI (kg/m^2^)	24.2 ± 3.4
NRS	7.6 ± 1.7
Testicular volume (mL)	15.2 ± 4.4
Varicocele location	
Right	2 (5%)
Bilateral	12 (27%)
Varicocele grade	
I	7 (16%)
II	23 (52%)
III	14 (32%)
Semen analysis quality	
Normal	24 (55%)
Oligozoospermia	1 (2%)
Asthenozoospermia	16 (36%)
OAT syndrome	3 (7%)

BMI = body mass index, BW = body weight, NRS = numeric rating scale, OAT = oligospermia, asthenozoospermia, teratozoospermia.

### 2.1. Pain assessment

The presence of pain was recorded subjectively the first time the patient visited our out-patient clinic and at the post-operative follow-up (post-operative day 7). The pain level was rated using an NRS from 0 (painless) to 10 (the most pain ever experienced). Pain resolution was defined as remission of symptoms such as pain or no further treatment required 3 months after the operation, while pain resolution failure was defined as persistent symptoms and analgesic drugs needed after the operation within a 3-month follow-up period.

### 2.2. Sperm analysis

We chose to only evaluate sperm volume, progressive motility, non-progressive motility, sperm concentration and morphology, since other parameters have been shown to have no significant effect according to previous literature.^[13]^ We used reference values of seminal quality established by the World Health Organization in 2010. The patient sperm was graded according to its motility; Grade A – rapid progressive motility, Grade B – slow or sluggish progressive motility, Grade C – nonprogressive motility, and Grade D – immotile. The minimum sperm concentration was 15 × 10^6^ per mL; ≥4% of the morphologically normal forms were above the lower reference limit.

### 2.3. Testicular volume

Testicular size was measured using an ultrasonography tool, which measured 3 ipsilateral testicle dimensions. The volume of the testicle was established by multiplying the dimensions by the standard coefficient of 0.52.

### 2.4. Surgical technique

All patients underwent subinguinal microsurgical varicocelectomy by an experienced andrologist.^[14,15]^ A horizontal 2 to 3 cm subinguinal incision was made below the external ring. The Camper and Scarpa fascia were then divided by means of electrocautery. Blunt dissection using the surgeon index finger was performed distally and proximally along the cord, deep to the Scarpa fascia, following which the cord could be easily grasped with a Babcock clamp. After that, the operating microscope was brought into the field. Under 10-power magnification, the external and internal spermatic fascia were opened. The veins were seen clearly under the microscope. All veins within the cord, except for the vasal veins, were doubly ligated by passing 2 6-0 Prolene ligatures beneath the vein. After the dissection, only the testicular arteries, cremasteric arteries, cremaster muscle fibers, nerves, lymphatic vessels, and vas deferens with its vessels remained.

### 2.5. Statistical analysis

SPSS version 22.0 was used for all statistical analyses. The demographic data were presented as frequency, mean and standard deviation. The clinical outcomes, including sperm analysis before and after the operation, were compared using a paired Student *t* test. Primary outcomes were evaluated using multivariate analysis and area under the receiver operating characteristic (ROC) curve. *P* < .05 was considered to indicate a statistically significant difference.

## 3. Results

Forty-four patients were included in the data analysis. The patients’ mean age was 33.3 ± 8.5 years, and the average preoperative NRS score was 7.6 ± 1.7. Other demographic characteristics of the study population are shown in Table 1. The microsurgical varicocelectomy outcomes are shown in Table 2. The mean number of veins ligated during the operations was 6.6 ± 2.7. The mean NRS on postoperative day 7 was 1.5 ± 1.1. The change in NRS (postoperative day 7 vs preoperative) was 6.1 ± 2.1, and the satisfaction score after surgery was 9.2 ± 1.1 according to the patient subjective feelings. The overall pain resolution rate was 91%. Four patients had postoperative complications, including 1 with chronic scrotal pain after the operation. Other patients had minor complications, including 1 with hydrocele which was found by sonography, and 2 with mild wound infections; both were grade 1 complications according to the Clavien-Dindo classification system. The wound infections subsided after the patients were educated about cleaning the wound and after they had taken oral antibiotics for 7 days.

**Table 2:** Surgical outcomes after microsurgical varicocelectomy.

Characteristic	Value
Total operation time (min)	102.6 ± 39.6
The number of veins ligated	6.6 ± 2.7
POD-7 NRS	1.5 ± 1.1
Changes in NRS	6.1 ± 2.1
Post-op satisfaction	9.2 ± 1.1
Pain resolution rate (%)	91
Complication rate (%)	9

NRS = numeric rating scale, POD = post-operative day.

Table 3 shows comparisons of the preoperative and postoperative semen parameters. The results showed that there was no significant improvement after microsurgical varicocelectomy at 3 months follow-up.

**Table 3:** Pre- and post-operative semen parameters.

			Pre-op	Post-op	*P* value
Sperm analysis parameters (N = 14)	Volume (mL)		3.5 ± 1.7	3.2 ± 1.3	.397
	Motility (%)	Progressive, PR	24.6 ± 15.0	25.3 ± 13.5	.903
		Non progressive, NP	17.3 ± 11.0	18.4 ± 13.4	.821
		Immotile	50.9 ± 21.7	42.0 ± 20.6	.092
	Sperm Count (x100,000/mL)		374.3 ± 445.6	402.2 ± 276.3	.813
	Morphology (%)	Normal	41.5 ± 22.8	52.3 ± 27.5	.233

**Figure 2.**
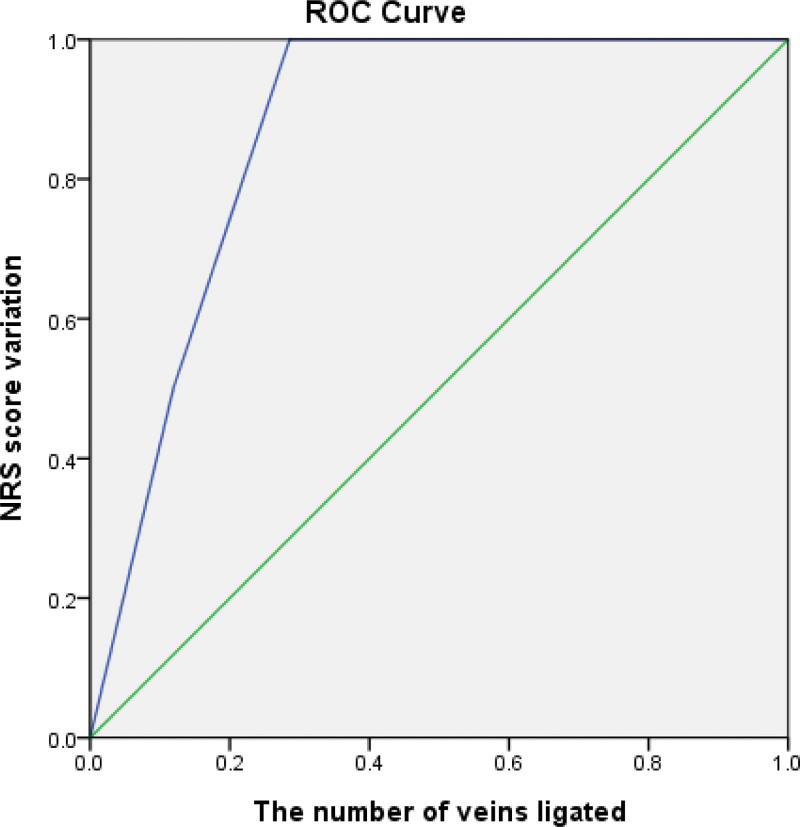
Area under ROC curve for changes in NRS score and total number of veins ligated. NRS = numeric rating scale, ROC = receiver operating characteristic curve.

Multivariate analysis revealed that severe varicocele grade (odds ratio [OR] 16.5, 95% confidence interval [CI] 3.01–90.47) and the number of veins ligated (OR 6, 95% CI 1.6–22.48) were significantly associated with changes in the NRS after surgery (Table 4). The area under the ROC curve for changes in NRS and the total number of veins ligated was 0.869 (Fig. 2).

**Table 4:** Multivariate analysis of pre-operative and intraoperative parameters of determinants associated with NRS score variation.

Parameter	*P* value	OR (95% CI)
Age	.609	-
BMI	.862	-
Varicocele grading	**.018** ***	**16.5 (3.01–90.47**)
Testicular volume	.566	-
SA volume	.64	-
SA PR	.437	-
SA NP	.063	-
SA immotile	.378	-
SA sperm count	.993	-
SA normal	.783	-
Operation time	.491	-
The number of veins ligated	**.013** ***	**6 (1.6–22.48**)

Bold values indicates indicative ofthepotential for improved pain outcomes in patients with a preoperative diagnosis of severe varicocele, or patients whoundergo intraoperative ligation of a greater number of veins.

BMI = body mass index, NP = non-progressive, NRS = numeric rating scale, PR = progressive, SA = semen analysis.

*** *P* < .05

## 4. Discussion

The effect of varicocele on male infertility is well documented. However, the extent to which it contributes to scrotal pain is still not well understood. In this study, more of the patients who visited our clinic had varicocele pain than male infertility, and this was the rationale for conducting the study. Many scrotal and non-scrotal conditions have the potential to manifest as scrotal pain, including chronic pelvic pain syndrome, recent testicular or groin trauma, testicular malignancy, benign testicular masses, hydroceles, spermatoceles, epididymal cysts, inguinal hernias, post-surgery pain, varicocele, and infection. When conservative measures do not successfully relieve symptoms of a varicocele, surgery is recommended. Shridharani et al summarized the pain resolution success rate according to different surgical approaches. Among patients who underwent a microsurgical technique, approximately 85% achieved complete resolution of pain, 6% achieved partial resolution, and 9% had no improvement in pain resolution after the surgery.^[16]^ In our study, our pain resolution rate was 91%, and only 1 person had chronic scrotal pain during follow-up, which is comparable with previous literature.^[16]^

Researchers have also investigated additional predictive factors for surgical success in effectively alleviating post-surgery pain. Shindel et al found that younger age and increased varicocele grade were both predictive of varicocele pain.^[17]^ In addition, Kim et al reported a noteworthy correlation between the nature of pain experienced and the success rate following varicocelectomy. When the pain was described as dull rather than dragging in nature, the resolution of pain was reported to be 100% complete.^[6]^ In addition, Altunoluk et al reported that patients with a longer duration of pain prior to surgery exhibited a higher success rate (98.6%) compared to patients with a shorter duration of pain (17.7%).^[9]^ Therefore, both the nature and duration of pain may be independent predictive factors for the successful resolution of post-surgical pain. A prior study found that high-grade varicocele might predict the likelihood of pain resolution after surgery,^[18]^ which is consistent with our findings. These results align with the notion that more significant vein dilation may cause more severe heaviness in the scrotum, and therefore more varicocele-associated pain. Dilation of veins to a higher degree may also worsen pain mechanisms, such as discomfort from toxic metabolite reflux and neural fiber compression due to dilated veins.^[19,20]^ On the other hand, however, Park et al found the opposite results in that varicocele grade was not correlated with pain resolution.^[21]^ Consequently, further comprehensive studies are warranted to elucidate this issue in future research.

The effect of the number of veins intraoperatively ligated has seldom been investigated in terms of pain resolution after surgery. The correlation between the extent of vein ligation during surgery and semen analysis outcomes was assessed by Shindel et al, who found a notable positive association between the number of veins ligated and the overall motility of sperm (*P* = .017).^[22]^ The variability in the number of veins encountered during surgical procedures is linked to the clinical grade of varicocele. It is therefore compatible with the theory that the more veins encountered, the more testicular congestion would exist, and consequently the more scrotal pain would be caused. However, Elbardisi et al determined that while a significant improvement in postoperative pain was detected in patients undergoing microsurgical subinguinal varicocelectomy, the number of veins ligated intraoperatively did not have a significant effect (*P* = .81).^[23]^ This finding is in contrast to the results of our study. However, in their study, they categorized the patients into 3 groups according to the number of spermatic veins ligated during varicocelectomy (<5 veins, 5–10 veins and >10 veins), while we analyzed the exact number of veins ligated during the operation. Our results indicated that varicocele grade and varicocele vein ligation were correlated with pain after all other parameters were eliminated. In addition, the area under the ROC curve for changes in NRS and the total number of veins ligated showed that they were significantly predictive factors (0.869), meaning that the number of veins ligated was a strong predictor of changes in the NRS. Possible reasons for this difference could be because all of our patients received microsurgical varicocelectomy by the same experienced andrologist using a delicate surgical technique. In addition, we used the exact number of ligated veins instead of categorizing the participants into different groups.^[23]^ Furthermore, we recorded changes in pain according to the NRS, while the previous study only recorded whether there was pain improvement or not.

As for semen quality, many studies in recent years have shown that microscopic varicocelectomy can improve semen quality. However, we found no significant improvement after microsurgical varicocelectomy at 3 months of follow-up (Table 3). The reason for this discrepancy in our results compared to most previous studies could be due to the fact that our patients were specifically enrolled for pain management, and their initial semen analysis was not significantly impaired. Therefore, the semen quality improvement after microsurgical varicocelectomy was not as obvious as those who received the operation due to male infertility. In addition, the process of spermatogenesis takes approximately 74 to 120 days,^[24,25]^ and most of our patient received semen analysis at 3 months of follow-up due to health insurance considerations. If we had extended the follow-up period for up to 1 year postoperatively, we believe that the results may have been different.

Our study has several limitations. First, this study included a relatively few number of patients and it was retrospective in design. In order to ensure homogeneity, we only analyzed a single experienced andrologist who was trained in microsurgery for male infertility at Weill Cornell Medicine in New York, United States. As a result, the number of enrolled patients was limited. Second, the pain assessment scale was used subjectively, which may have led to bias in the patient judgment of the intensity and quality of their pain, as every patient tolerance to pain can differ. Finally, we evaluated semen quality after a relatively short time after surgery due to health insurance considerations.

## 5. Conclusion

Microsurgical varicocelectomy had a high success rate for scrotal pain and satisfaction. Severe varicocele grade and the number of veins ligated in microsurgical varicocelectomy were associated with postoperative pain improvement.

## Author contributions

**Conceptualization:** Chun-Yo Laih, Chi-Ping Huang.

**Data curation:** Wei-Chun Huang.

**Formal analysis:** Wei-Chun Huang, Fang-Yu Ku.

**Investigation:** Fang-Yu Ku.

**Methodology:** Chun-Ming Lai, Yun-Yi Wang.

**Project administration:** Chun-Yo Laih, Chi-Ping Huang, Yun-Yi Wang.

**Resources:** Chun-Yo Laih, Fang-Yu Ku.

**Software:** Chun-Ming Lai, Hsu-Ning Hsu.

**Supervision:** Chao-Tung Yang, Chun-Yo Laih.

**Validation:** Hsu-Ning Hsu.

**Visualization:** Chao-Tung Yang, Yun-Yi Wang.

**Writing – original draft:** Wei-Chun Huang.

**Writing – review & editing:** Wei-Chun Huang, Chun-Yo Laih.

## References

[1] Lomboy JR Coward RM. The varicocele: clinical presentation, evaluation, and surgical management. Semin Intervent Radiol. 2016;33:163–9. 27582602 10.1055/s-0036-1586143 PMC5005075

[2] Paick S Choi WS. Varicocele and testicular pain: a review. World J Mens Health. 2019;37:4–11. 29774668 10.5534/wjmh.170010 PMC6305863

[3] Khera M Lipshultz LI. Evolving approach to the varicocele. Urol Clin North Am. 2008;35:183–9, viii. 18423239 10.1016/j.ucl.2008.02.001

[4] Marmar JL Kim Y. Subinguinal microsurgical varicocelectomy: a technical critique and statistical analysis of semen and pregnancy data. J Urol. 1994;152:1127–32. 8072081 10.1016/s0022-5347(17)32521-1

[5] Abrol N Panda A Kekre NS. Painful varicoceles: role of varicocelectomy. Indian J Urol. 2014;30:369–73. 25378815 10.4103/0970-1591.128497 PMC4220373

[6] Kim HT Song PH Moon KH. Microsurgical ligation for painful varicocele: effectiveness and predictors of pain resolution. Yonsei Med J. 2012;53:145–50. 22187245 10.3349/ymj.2012.53.1.145 PMC3250320

[7] Abd EM Asker W Abbas A. Varicocelectomy to treat pain, and predictors of success: a prospective study. Curr Urol. 2012;6:33–6. 24917707 10.1159/000338867 PMC3783314

[8] Kim SO Jung H Park K. Park K Outcomes of microsurgical subinguinal varicocelectomy for painful varicoceles. J Androl. 2012;33:872–5. 22174387 10.2164/jandrol.111.014993

[9] Altunoluk B Soylemez H Efe E. Duration of preoperative scrotal pain may predict the success of microsurgical varicocelectomy. Int Braz J Urol. 2010;36:55–9. 20202236 10.1590/s1677-55382010000100009

[10] Karademir K Senkul T Baykal K. Evaluation of the role of varicocelectomy including external spermatic vein ligation in patients with scrotal pain. Int J Urol. 2005;12:484–8. 15948749 10.1111/j.1442-2042.2005.01063.x

[11] Park HJ Lee SS Park NC. Predictors of pain resolution after varicocelectomy for painful varicocele. Asian J Androl. 2011;13:754–8. 21102471 10.1038/aja.2010.87 PMC3739586

[12] Dubin L Amelar RD. Varicocele size and results of varicocelectomy in selected subfertile men with varicocele. Fertil Steril. 1970;21:606–9. 5433164 10.1016/s0015-0282(16)37684-1

[13] Pajovic B Radojevic N Dimitrovski A. Advantages of microsurgical varicocelectomy over conventional techniques. Eur Rev Med Pharmacol Sci. 2015;19:532–8. 25753866

[14] Goldstein M Gilbert BR Dicker AP. Microsurgical inguinal varicocelectomy with delivery of the testis: an artery and lymphatic sparing technique. J Urol. 1992;148:1808–11. 1433614 10.1016/s0022-5347(17)37035-0

[15] Marmar JL Kim Y. Subinguinal microsurgical varicocelectomy: a technical critique and statistical analysis of semen and pregnancy data. J Urol. 1994;152:1127–32. 8072081 10.1016/s0022-5347(17)32521-1

[16] Shridharani A Lockwood G Sandlow J. Varicocelectomy in the treatment of testicular pain: a review. Curr Opin Urol. 2012;22:499–506. 22965318 10.1097/MOU.0b013e328358f69f

[17] Punjani N Wald G Gaffney CD. Predictors of varicocele-associated pain and its impact on semen parameters following microsurgical repair. Andrologia. 2021;53:e14121. 34118088 10.1111/and.14121

[18] Kim SO Jung H Park K. Outcomes of microsurgical subinguinal varicocelectomy for painful varicoceles. J Androl. 2012;33:872–5. 22174387 10.2164/jandrol.111.014993

[19] Lundy SD Sabanegh ES Jr Varicocele management for infertility and pain: A systematic review. Arab J Urol. 2017;16:157–70. 29713547 10.1016/j.aju.2017.11.003 PMC5922006

[20] Paick S Choi WS. Varicocele and testicular pain: a review. World J Mens Health. 2019;37:4–11. 29774668 10.5534/wjmh.170010 PMC6305863

[21] Park JH Pak K Park NC. How can we predict a successful outcome after varicocelectomy in painful varicocele patients? An updated meta-analysis. World J Mens Health. 2021;39:645–53. 32009313 10.5534/wjmh.190112 PMC8443985

[22] Shindel AW Yan Y Naughton CK. Does the number and size of veins ligated at left-sided microsurgical subinguinal varicocelectomy affect semen analysis outcomes? Urology. 2007;69:1176–80. 17572210 10.1016/j.urology.2007.01.086

[23] Elbardisi H Agarwal A Majzoub A. Does the number of veins ligated during microsurgical subinguinal varicocelectomy impact improvement in pain post-surgery? Transl Androl Urol. 2017;6:264–70. 28540234 10.21037/tau.2017.03.56 PMC5422682

[24] Amann RP. The cycle of the seminiferous epithelium in humans: a need to revisit? J Androl. 2008;29:469–87. 18497337 10.2164/jandrol.107.004655

[25] Forster P Hohoff C Dunkelmann B. Elevated germline mutation rate in teenage fathers. Proc Biol Sci. 2015;282:20142898. 10.1098/rspb.2014.2898 PMC4345458 25694621

